# Resting Metabolic Rate and Lung Function in Wild Offshore Common Bottlenose Dolphins, *Tursiops truncatus*, Near Bermuda

**DOI:** 10.3389/fphys.2018.00886

**Published:** 2018-07-17

**Authors:** Andreas Fahlman, Katherine McHugh, Jason Allen, Aaron Barleycorn, Austin Allen, Jay Sweeney, Rae Stone, Robyn Faulkner Trainor, Guy Bedford, Michael J. Moore, Frants H. Jensen, Randall Wells

**Affiliations:** ^1^Fundación Oceanografic de la Comunidad Valenciana, Gran Vía Marques del Turia, Valencia, Spain; ^2^Department of Life Sciences, Texas A&M University–Corpus Christi, Corpus Christi, TX, United States; ^3^Woods Hole Oceanographic Institution, Woods Hole, MA, United States; ^4^Chicago Zoological Society’s Sarasota Dolphin Research Program, Mote Marine Laboratory, Sarasota, FL, United States; ^5^Duke University Marine Lab, Beaufort, NC, United States; ^6^Dolphin Quest, Waikoloa, HI, United States; ^7^Wildlife Consulting Service, Currumbin, QLD, Australia; ^8^Aarhus Institute of Advanced Studies, Aarhus University, Aarhus, Denmark

**Keywords:** lung mechanics, total lung capacity, field metabolic rate, energetics, minimum air volume, diving physiology, marine mammals, spirometry

## Abstract

Diving mammals have evolved a suite of physiological adaptations to manage respiratory gases during extended breath-hold dives. To test the hypothesis that offshore bottlenose dolphins have evolved physiological adaptations to improve their ability for extended deep dives and as protection for lung barotrauma, we investigated the lung function and respiratory physiology of four wild common bottlenose dolphins (*Tursiops truncatus*) near the island of Bermuda. We measured blood hematocrit (Hct, %), resting metabolic rate (RMR, l O_2_ ⋅ min^-1^), tidal volume (*V*_T_, l), respiratory frequency (*f*_R_, breaths ⋅ min^-1^), respiratory flow (l ⋅ min^-1^), and dynamic lung compliance (*C*_L_, l ⋅ cmH_2_O^-1^) in air and in water, and compared measurements with published results from coastal, shallow-diving dolphins. We found that offshore dolphins had greater Hct (56 ± 2%) compared to shallow-diving bottlenose dolphins (range: 30–49%), thus resulting in a greater O_2_ storage capacity and longer aerobic diving duration. Contrary to our hypothesis, the specific *C*_L_ (s*C*_L_, 0.30 ± 0.12 cmH_2_O^-1^) was not different between populations. Neither the mass-specific RMR (3.0 ± 1.7 ml O_2_ ⋅ min^-1^ ⋅ kg^-1^) nor *V*_T_ (23.0 ± 3.7 ml ⋅ kg^-1^) were different from coastal ecotype bottlenose dolphins, both in the wild and under managed care, suggesting that deep-diving dolphins do not have metabolic or respiratory adaptations that differ from the shallow-diving ecotypes. The lack of respiratory adaptations for deep diving further support the recently developed hypothesis that gas management in cetaceans is not entirely passive but governed by alteration in the ventilation-perfusion matching, which allows for selective gas exchange to protect against diving related problems such as decompression sickness.

## Introduction

Marine mammals live a life of dual constraints: On one hand, they need to find and exploit prey in varying densities underwater, but on the other hand as air breathing animals they need to return to the surface to replenish O_2_ stores and remove CO_2_ produced by aerobic metabolism. The total time spent on a single dive influences both the types of prey that can be exploited as well as the efficiency with which food can be obtained, and this dive performance ultimately depends on the amount of O_2_ available, the size of the O_2_ stores, and how quickly they are utilized, the metabolic cost.

To increase dive capacity, many marine mammals have therefore evolved to lower energy consumption over the dive and to increase available O_2_ stores. Oxygen stores are typically expanded by having a greater proportion of blood relative to body mass, by increasing the amount of red blood cells per volume of blood (the hematocrit), and by increasing the amount of myoglobin (Ponganis, 2015). In addition, it has been proposed that deep divers have relatively larger muscle mass, large muscle fibers, and low mitochondrial volume (Kooyman and Ponganis, 1998; Pabst et al., 2016). A greater proportion of blood volume and muscle mass would enhance available O_2_, and the latter would also help to reduce the overall rate of O_2_ consumption as the basal metabolic rate of muscle is lower than many other tissues (Pabst et al., 2016). The lung volume, on the other hand, does not seem to be increased, and it has been proposed that deep diving species have lungs that are relatively small as compared with shallow diving species (Scholander, 1940; Piscitelli et al., 2010). It is suggested that the reduced lung volume minimizes gas exchange at depth and minimizes uptake of N_2_ and thereby the risk of diving-related problems such as decompression sickness or N_2_ narcosis (Scholander, 1940; Kooyman, 1973; Bostrom et al., 2008).

In addition to reduced lung volume in deep divers, it has been suggested that marine mammals have respiratory adaptations that help prevent excessive uptake of N_2_. The respiratory adaptations include a highly compliant alveolar space and stiff airways that would allow the alveolar gas to empty into the conducting airways as the pressure increases during descent (Scholander, 1940). In addition, the minimum air volume (MAV), the amount of air left in the relaxed lung, would be minimal to avoid barotrauma (Fahlman et al., 2017b). In a recent study, it was shown that the static lung compliance in pinnipeds that had been housed under human care was lower as compared with wild animals undergoing rehabilitation (Fahlman et al., 2014). It was hypothesized that these differences could be evidence that lung conditioning, where repeated collapse and recruitment of the alveoli, increased the compliance (Fahlman et al., 2014). However, the study subjects in the previous study were of different ages, species, and health and we are unaware of any study that has specifically tested the hypothesis that dive behavior alters lung compliance.

In the current study, we wanted to test the hypothesis that offshore bottlenose dolphins have lower metabolic rate, and tidal volume (*V*_T_), and greater lung compliance (*C*_L_) as traits that correlate with deep diving. To test these hypotheses, we measured the resting metabolic rate (RMR), *V*_T_, respiratory frequency (*f*_R_), respiratory flow, and dynamic *C*_L_ in 4 common bottlenose dolphins near the island of Bermuda. The dolphins were of the offshore ecotype (Wells and Scott, 2018), a variant occupying deeper, offshore waters, and differing from the more commonly studied coastal ecotype in distribution and morphology (Mead and Potter, 1995). Previous research found that offshore dolphins near Bermuda exploited prey at depths of several hundred meters in dives lasting more than 5 min, well beyond the depths and dive durations previously recorded for coastal bottlenose dolphins (Klatsky et al., 2007). Our aim was to compare these measurements with data from coastal ecotype bottlenose dolphins (Fahlman et al., 2018a) to test the hypothesis that deeper-diving offshore ecotype bottlenose dolphins have a lower mass-specific metabolic rate, a lower *V*_T_, and a greater *C*_L_ as part of traits to improve the ability for extended deep dives and as protection against lung barotrauma.

## Materials and Methods

### Animals

A total of 3 male and 1 female common bottlenose dolphins (*Tursiops truncatus*, **Table 1**) were studied. The animals were captured by a break-away hoop netting technique (Asper, 1975), and briefly restrained for a health assessment, tagging, and sampling. Once in the net, swimmers managed the animal and moved it onto a buoyant foam mat. The animal was moved onto a sling and then brought onto the sampling boat using a pulley system. Once on the boat, the dolphin was weighed (± 0.2 kg, Ohaus 3000 Series industrial floor scale) and underwent a basic health examination (e.g., Wells et al., 2004). During the health assessment, a custom-made breath-by-breath respirometry system, including a custom-built pneumotachometer and fast-response O_2_ and CO_2_ analyzers allowed assessment of lung function and resting rates of O_2_ consumption ($\dot{V}$O_2_) and CO_2_ production ($\dot{V}$CO_2_). All spirometry trials (breath-by-breath lung function and end-expired O_2_ and CO_2_) were measured with the dolphin lying on a shaded, padded mat or being lightly restrained while partially submerged in water and breathing voluntarily. Spirometry trials while resting on the foam mat ranged from 35 to 45 min, and while restrained in water for up to 10 min.

**Table 1:** Animal identification, sex (M-male, F-female), body mass (*M*_b_), straight length (SL), maximum girth (G), blood hematocrit concentration (Hct, %), number of breaths collected (N), and breathing frequency (*f*_R_), while in air or in water.

Animal ID	Sex	*M*_b_ (kg)	SL (cm)	G (cm)	[Hct]	N		*f*_R_ (breaths ⋅ min^-1^)	
							
						Air	Water	Air	Water
Tt19	M	294	256	142	54	104	17	3.9	4.7
Tt20	M	282	261	142	54	222	29	5.8	9.7
Tt21	F	173	238	144	57	166	33	6.1	9.6
Tt22	M	282	251	144	59	127	24	4.4	3.0
Mean		258 ± 57	252 ± 10	143 ± 1	56 ± 2	155 ± 52	26 ± 7	5.1 ± 1.0	6.7 ± 3.4


All works were approved by the IACUC at Texas A&M University Corpus Christi (TAMUCC-IACUC AUP#04-11) and by a research permit issued by the Bermuda Government, Department of Environment and Natural Resources (Research permit number SP160401r).

### Blood Hematocrit (Hct)

Blood samples were obtained from the venous rete on the ventral side of the tail fluke for measuring the packed cell volumes, or blood hematocrit (Hct).

### Respiratory Flows (Lung Function)

The procedures and equipment used were identical to those used in previous studies on the same species under human care, or during similar conditions on restrained wild dolphins (Fahlman et al., 2015, 2018a), briefly summarized below. Respiratory flows were measured using a custom-made Fleisch type pneumotachometer (Mellow Design, Valencia, Spain), which housed a low-resistance laminar flow matrix (Item # Z9A887-2, Merriam Process Technologies, Cleveland, OH, United States). A diffuser was inserted to improve the flow characteristics during expiration, which reduced the need for different calibration factors for expiratory and inhalatory flow. A differential pressure transducer (Spirometer Pod, ML 311, ADInstruments, Colorado Springs, CO, United States) was connected to the pneumotachometer with two, 310 cm lengths of 2 mm i.d., firm walled, flexible tubing. The differential pressure transducer was connected to a data acquisition system (Powerlab 8/35, ADInstruments, Colorado Springs, CO, United States), and the data were captured at 400 Hz and displayed on a computer running LabChart (v. 8.1, ADInstruments, Colorado Springs, CO, United States). The differential pressure was used to determine flow and was calibrated using a 7.0 l calibration syringe (Series 4900, Hans Rudolph, Inc., Shawnee, KS, United States). The signal was integrated and the flow determined as detailed previously (Fahlman et al., 2015, 2018a).

### Lung Compliance

We estimated the dynamic lung compliance (*C*_L_, l ⋅ cmH_2_O^-1^) as previously described by dividing the *V*_T_ by the change in transpulmonary pressure (P_L_ = P_ao_ – P_eso_; P_ao_ = airway opening pressure, P_eso_ = esophageal pressure), measured at zero flow of the expiratory phase and inspiratory phases (a and b, respectively, in Figure 2C in Fahlman et al., 2015). To enable comparisons between animals of different body sizes, we computed the mass-specific lung compliance (s*C*_L_, cmH_2_O^-1^) by dividing *C*_L_ by the MAV, estimated to be 7% of the estimated total lung capacity (TLC; Fahlman et al., 2011, 2015).

To learn more about the respiratory adaptations in marine mammals, we measured trans-pulmonary and trans-thoracic static compliance of a stranded and deceased common (*Delphinus delphis*) and Atlantic white-sided dolphin (*Lagenorhynchus acutus*) obtained from the Marine Mammal Rescue and Research Division of the International Fund for Animal Welfare (IFAW). The specimen was intubated and the transthoracic pressure of the intact cadaver (P_T_ = P_ao_ - P_amb_, P_amb_ = ambient pressure) or the P_L_ of the excised lungs (P_L_ = P_ao_ - P_amb_) as previously detailed (Fahlman et al., 2011, 2014). These former value represent the compliance of the entire respiratory system, including lung and chest, and the latter the compliance of the lungs only. If the chest has high compliance, P_L_ = P_T_.

### Respiratory Gas Composition

The concentrations of expired O_2_ and CO_2_ were subsampled via a port in the pneumotachometer and passed through a 310 cm length of 2 mm i.d., firm walled, flexible tubing and a 30 cm length of 1.5 mm i.d., Nafion tubing, to a fast-response O_2_ and CO_2_ analyzer (Gemini respiratory monitor, CWE Inc.) at a flow rate of 200 ml ⋅ min^-1^. The gas analyzer was connected to the data acquisition system and sampled at 400 Hz. The gas analyzer was calibrated before and after the experiment using a commercial mixture of 5% O_2_, 5% CO_2_, and 90% N_2_ (Product No. 17L-340, GASCO, Oldsmar, FL, United States). Ambient air and pure O_2_ were used to check the calibration before and after each experimental trial. Mean (± SEM) air temperature and humidity during trials were 31.3 ± 0.3°C (*n* = 4, range 31–32°C) and 71.8 ± 3.6% (65–78%). The average ( ± SE, *n* = 4) surface water temperature around the sampling boat was 27.3 ± 0.3°C (range: 27–28°C).

### Metabolic Rates

The metabolic rates were estimated as detailed previously (Fahlman et al., 2015, 2018a) and briefly summarized here. The respiratory gas signals were phase-corrected for CO_2_ and O_2_, to match the respirations, and the expiratory flow-rate and expired O_2_ and CO_2_ content were multiplied to calculate the instantaneous O_2_ consumption rate ($\dot{V}$O_2_) and CO_2_ production rate ($\dot{V}$CO_2_). The instantaneous $\dot{V}$O_2_ and $\dot{V}$CO_2_ were integrated over each breath to yield the total volume of O_2_ and CO_2_ exchanged during each breath. The volumes were summed for each trial period and divided by the duration of the trial to provide an estimate of the $\dot{V}$O_2_ and $\dot{V}$CO_2_ rates for that time period.

We compared metabolic rate in water and on land to determine if the effect of gravity would affect the measurements. Measurements on land are easier to perform during health assessments, but in fully aquatic species, the effect of gravity on respiration may increase the metabolic cost. However, we found no evidence for this in the current study. Still, comparisons with the coastal ecotype were made with the in-water measurements.

### Data Processing and Statistical Analysis

All gas volumes were converted to standard temperature pressure dry (STPD, Quanjer et al., 1993). Exhaled air was assumed saturated at 37°C, inhaled air volume was corrected for ambient temperature and relative humidity. Metabolic data are reported as the average O_2_ consumption rate for an entire trial. With the limited sample size, we made comparisons between positions (air vs. water) or respiratory direction (inspiration vs. expiration) using paired *t*-tests. In this study, *P*-values ≤ 0.05 were considered as significant and *P* ≤ 0.1 were considered potentially suggestive of a trend. Data are presented as the mean ± standard deviation (SD), unless otherwise stated.

## Results

Data from 722 spontaneous breaths were collected for 4 bottlenose dolphins (**Table 1**) sampled near the island of Bermuda.

### Blood Hematocrit (Hct)

The blood Hct ranged from 54 to 59% between animals and are reported in **Table 1**.

### Respiratory Flows and Timing

The average *f*_R_ during trials was 5.9 ± 2.2 breaths ⋅ min^-1^ (**Table 1**), and did not differ whether held in or out of water (paired *t*-test, *t*-value: 1.37, df = 3, *P* = 0.26).

The average maximum spontaneous inspiratory flow ($\dot{V}$_insp_) was 16.1 ± 3.6 l ⋅ s^-1^, and was not different for animals in air vs. in water (range: 2.5–34.2 l ⋅ s^-1^, paired *t*-test, *t*-value: 0.79, df = 3, *P* = 0.49). Similarly, the maximum spontaneous expiratory flow ($\dot{V}$_exp_) did not differ in water vs. in air (21.0 ± 5.2 l ⋅ sec^-1^, range: 7.5–39.4 l ⋅ s^-1^, paired *t*-test, *t*-value: 1.5, df = 3, *P* = 0.24). $\dot{V}$_exp_ was significantly higher as compared with $\dot{V}$_insp_ (paired *t*-test, *t*-value: 3.2, df = 3, *P* = 0.04). The average expiratory (e.g., 514 ± 109 s vs. 458 ± 110 s; paired *t*-test, *t*-value: 4.66, *P* = 0.02), average inspiratory (*t*-value: 3.47, *P* = 0.04), and total breath durations (*t*-value: 5.17, *P* = 0.01) were shorter in water as compared with air (**Table 2**). The average expiratory or inspiratory breath duration did not differ either in air or water (e.g., 514 ± 109 s vs. 653 ± 209 s; air: *t*-value: 2.4, df = 3, *P* = 0.1, water: *t*-value: 1.2, df = 3, *P* = 0.31). The expiratory (*t*-value: 1,25, df = 3, *P* = 0.30) or inspiratory *V*_T_ (*t*-value: 0.89, df = 3, *P* = 0.43) did not differ in air vs. water, and there were no differences in inspiratory *V*_T_ (*V*_Tinsp_ = 6.0 ± 0.8 l) or expiratory *V*_T_ (*V*_Texp_ = 5.5 ± 0.9 l, *t*-value: 1.28, df = 3, *P* = 0.29). The average *V*_Tinsp_ was 27 ± 1% of the estimated total lung capacity (TLC_est_, 8).

**Table 2:** Animal identification, expiratory (*T*_exp_), inspiratory (*T*_insp_) and total breath duration (*T*_tot_), expiratory and inspiratory flow ($\dot{V}$_exp_ and $\dot{V}$_insp_) and tidal volume (*V*_Texp_ and *V*_Tinsp_), specific dynamic lung compliance (s*C*_L_).

Animal ID	*T*_exp_	*T*_insp_	*T*_tot_	$\dot{V}$_exp_	$\dot{V}$_insp_	*V*_Texp_	*V*_Tinsp_	s*C*_L_
				
Air	ms			l ⋅ min^-1^		l		cmH_2_O^-1^
Tt19	548 ± 134	774 ± 134	1322 ± 217	20.4 ± 4.3	12.6 ± 2.9	6.4 ± 1.4	6.3 ± 1.4	0.56
Tt20	645 ± 275	885 ± 737	1530 ± 847	14.9 ± 10.2	12.9 ± 3.3	4.6 ± 2.9	5.7 ± 1.8	0.43
Tt21	470 ± 387	457 ± 106	927 ± 375	25.9 ± 6.4	19.4 ± 3.7	6.3 ± 2.6	6.3 ± 2.6	0.13
Tt22	391 ± 68	498 ± 87	889 ± 137	33.8 ± 11.1	22.8 ± 3.4	7.4 ± 2.2	7.7 ± 1.6	0.07
Mean	514 ± 109	653 ± 209	1167 ± 311	23.8 ± 8.1	16.9 ± 5.0	6.2 ± 1.2	6.5 ± 0.8	0.30 ± 0.23
**Water**								
Tt19	516 ± 59	647 ± 103	1164 ± 119	21.9 ± 3.8	13.6 ± 2.5	7.0 ± 1.7	6.7 ± 1.4	NA
Tt20	582 ± 223	598 ± 148	1216 ± 307	14.7 ± 11.2	15.3 ± 3.3	4.9 ± 4.2	6.8 ± 2.6	NA
Tt21	384 ± 77	354 ± 37	738 ± 90	16.4 ± 2.4	13.1 ± 2.2	3.5 ± 0.9	3.4 ± 0.8	NA
Tt22	349 ± 68	397 ± 87	746 ± 137	21.3 ± 11.1	19.4 ± 3.4	4.2 ± 2.2	5.4 ± 1.6	NA
Mean	458 ± 110	499 ± 145	966 ± 259	18.6 ± 3.6	15.4 ± 2.9	4.9 ± 1.6	5.6 ± 1.6	


### Respiratory Compliance

**FIGURE 1:**
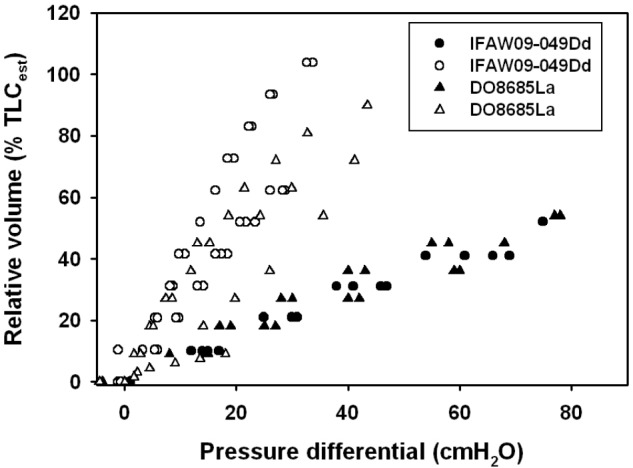
Lung volume expressed as a percentage of estimated total lung capacity (TLC_est_, Kooyman, 1973; Fahlman et al., 2011) vs. trans-thoracic (whole animals, filled symbols) or trans-pulmonary (excised, open symbols) pressure in a common (Dd: *Delphinus delphis*) and Atlantic white-sided dolphin (La: *Lagenorhynchus acutus*). Measurements shown are during deflation of the lung.

The average ( ± SEM) *C*_L_ and s*C*_L_ were 0.50 ± 0.22 l ⋅ cmH_2_O^-1^ (range: 0.12–0.98 l ⋅ cmH_2_O^-1^) and 0.30 ± 0.12 cmH_2_O^-1^ (range: 0.07–0.56 cmH_2_O^-1^), respectively. We used previously published pressure-volume (compliance) data for the excised lungs (Fahlman et al., 2011), and unpublished chest compliance data from the same individuals to compare differences in lung and chest compliance in carcasses of a common (*Delphinus delphis*) and an Atlantic white-sided dolphin (*Lagenorhynchus acutus*, **Figure 1**). These data show that the chest is not infinitely compliant and provide resistance to inhalation.

### Gas Exchange

The end-expiratory O_2_ was significantly higher in water as compared with air (*t*-value: 3.6, *P* = 0.04), and there was a suggestion of a trend for a lower end-expiratory CO_2_ in water as compared to on land (*t*-value: 2.9, *P* = 0.06, **Table 3**).

**Table 3:** Animal ID, end-expired CO_2_ and O_2_, CO_2_ production rate ($\dot{V}$CO_2_), O_2_ consumption rate ($\dot{V}$O_2_), and mass-specific $\dot{V}$O_2_ (s$\dot{V}$O_2_) while in air or in water.

Animal ID	CO_2_	O_2_	$\dot{V}$CO_2_	$\dot{V}$O_2_	s$\dot{V}$O_2_
				
Air	%		l CO_2_ ⋅ min^-1^	l O_2_ ⋅ min^-1^	ml O_2_ ⋅ min^-1^ ⋅ kg^-1^
Tt19	8.6 ± 0.7	12.1 ± 2.1	0.44	0.47	1.6
Tt20	6.4 ± 1.6	14.3 ± 2.8	0.70	0.68	2.4
Tt21	6.2 ± 1.5	14.3 ± 1.7	0.94	1.24	7.2
Tt22	6.5 ± 1.0	13.6 ± 2.1	0.31	0.45	1.6
Mean	6.9 ± 1.1	13.6 ± 1.1	0.60 ± 0.28	0.71 ± 0.37	3.2 ± 2.7
**Water**					
Tt19	7.0 ± 0.5	16.5 ± 1.1	0.73	0.85	2.9
Tt20	4.3 ± 1.5	18.6 ± 1.2	–	0.74	2.6
Tt21	4.4 ± 0.6	17.0 ± 0.9	0.58	0.68	3.9
Tt22	6.5 ± 1.0	14.4 ± 2.1	0.53	0.59	2.1
Mean	5.6 ± 1.4	16.7 ± 1.7	0.94 ± 0.67	0.72 ± 0.11	2.9 ± 0.8


### Metabolic Rates

The resting $\dot{V}$O_2_ was estimated for periods during which the dolphin remained calm that were at least 3 min and up to 15 min in duration (**Table 3**). Neither the $\dot{V}$O_2_ (*t*-value: 0.02, df = 3, *P* = 0.98), nor the $\dot{V}$CO_2_ (*t*-value: 0.24, df = 2, *P* = 0.83) differed on land vs. in water. The mass-specific metabolic rate (s$\dot{V}$O_2_) ranged from 1.6 to 7.2 ml O_2_ ⋅ min^-1^ ⋅ kg^-1^. A comparison with previous data from adult males showed that the s$\dot{V}$O_2_ in the offshore ecotype (2.53 ± 0.40 ml O_2_ ⋅ min^-1^ ⋅ kg^-1^, *n* = 3) did not differ with measurements in the near shore dolphins [2.69 ± 1.20 ml O_2_ ⋅ min^-1^ ⋅ kg^-1^, *n* = 9, (Fahlman et al., 2018a), *t*-test, *t*-value: 0.20, *P* = 0.85].

## Discussion

We estimated resting metabolic rate and lung function in four offshore bottlenose dolphins. This limited data set provides an important comparison with published values for animals under human care and free-ranging coastal bottlenose dolphins. We hypothesized that the deep-diving offshore population would have a lower s$\dot{V}$O_2_, s*V*_T_, and a higher s*C*_L_ as physiological adaptations required for deep-diving.

### Physiological Adaptations for Longer Foraging Dives

In the current study, the measured resting s$\dot{V}$O_2_ was within the same range as values previously published for bottlenose dolphins of the coastal ecotype (**Table 3**; Yazdi et al., 1999; Yeates and Houser, 2008; Noren et al., 2013; Fahlman et al., 2015, 2018a). The measured resting metabolic in free ranging coastal dolphins made in our previous study allowed intra-specific comparison of animals under similar conditions (Fahlman et al., 2018a). The O_2_ consumption rate of offshore dolphin males (range: 1.6–2.9 ml O_2_ ⋅ min^-1^ ⋅ kg^-1^) and the single female (3.9 ml O_2_ ⋅ min^-1^ ⋅ kg^-1^) while not different were at the lower end of values previously reported from both managed care and shallow-diving dolphins [0.8–9.5 ml O2 ⋅ min^-1^ ⋅ kg^-1^ (Yazdi et al., 1999; Yeates and Houser, 2008; Noren et al., 2013; Fahlman et al., 2015, 2018a)]. A lower metabolic rate would support the hypothesis that deep divers are built with a greater proportional muscle mass with large fibers and lower mitochondrial density (Pabst et al., 2016), which helps to increase the O_2_ stores and reducing the s$\dot{V}$O_2_, thereby increasing aerobic dive durations. However, we were unable to detect a significant difference in resting s$\dot{V}$O_2_ in adult males in water, which could be caused by the inability to control for digestive status or that the reduction is smaller than we could measure with our limited sample size. We had limited ability to control the sex, age, and size of the animal in the current study, but the goal was to select non-calf animals of medium size. We specifically avoided very young animals, and did not attempt to catch larger animals for safety reasons. Given that we were studying free-ranging animals, we could not determine their digestive status. As these variables affect the metabolic rate, this could have increased the variation of our measurements. Alternatively, the increased dive durations (average: 70 ± 10 s, maximum 539 s) seen in the offshore ecotype (Fahlman et al., 2018b) may be largely possible by increased O_2_ storage capacity.

We propose that the offshore ecotype has a greater O_2_ storage capacity to extend dive durations, and the greater blood Hct in the deep diving dolphins (Bermuda: 56 ± 2%) as compared with the shallow diving ecotype (values ranging from 37 to 47% for adult males and females) provide partial evidence of this (**Table 1**; Ridgway and Johnston, 1966; Hall et al., 2007; Klatsky et al., 2007; Schwacke et al., 2009). However, this greater blood Hct is not sufficient to increase the calculated aerobic dive limit (cADL) to support the longest dives seen in the Bermuda dolphins (Fahlman et al., 2018b). To further increase cADL, we previously proposed that the offshore ecotype have, in addition to reduced RMR and blood Hct, increased blood volume, muscle mass and muscle myoglobin as compared with the coastal ecotype (Pabst et al., 2016). A greater muscle and blood O_2_ capacity would make them less dependent on pulmonary O_2_ during deeper dives, which may explain the trend toward a lower mass-specific *V*_T_ (s*V*_T_). The longer and deeper dives performed by the offshore ecotype could allow them to further reduce the metabolic cost of foraging at depth by employing cost-saving swimming strategies that reduce the overall activity and metabolic cost (Williams, 2001; Fahlman et al., 2013; Martín López et al., 2015). Thus, offshore dolphins may have a reduced cost of foraging compared to coastal ecotype that increases their cADL. Comparing activity among these populations and ecotypes, as a proxy for field metabolic rate (acceleration, Fahlman et al., 2008, 2013; Enstipp et al., 2011) could be useful to assess how energy is partitioned in these populations.

### Breathing Strategies for Deep Foraging Dives

The measured *V*_T_ collected in this study agrees well with previous measurements in cetaceans (Fahlman et al., 2017b). The average s*V*_T_ recorded in the current study was 24.4 ± 2.6 ml ⋅ kg^-1^ (range: 21.1–32.1 ml ⋅ kg^-1^), which is within the range of previous estimates for the bottlenose dolphin (13.6–58.8 ml ⋅ kg^-1^, Irving et al., 1941; Ridgway et al., 1969; Fahlman et al., 2015, 2018a), and a gray whale (12.0–35.3 ml ⋅ kg^-1^, Wahrenbrock et al., 1974), but slightly lower than the harbor porpoise (39.3–52.6 ml ⋅ kg^-1^, Reed et al., 2000). The breathing frequency (5.1 ± 1.0 breaths ⋅ min^-1^ in air; 6.7 ± 3.4 breaths ⋅ min^-1^ in water), on the other hand, was significantly higher as compared with previous studies (Dolphin Quest: 3.4 ± 1.1 breaths ⋅ min^-1^, Sarasota: 2.9 ± 1.1 breaths ⋅ min^-1^, *t*-value > 2.9 for both, *P* < 0.05, *t*-test, Fahlman et al., 2015, 2018a). This variation may reflect differences in breathing strategy between deep- and shallow-diving dolphins. It is also possible that the differences in breathing frequency may indicate stress, where dolphins under managed care participate voluntarily and those in Sarasota have been handled previously.

The breathing strategy in marine mammals, with a low *f*_R_, high *V*_T_, and extended respiratory pause between inspirations has been hypothesized to help with gas exchange or aid in buoyancy (Mortola and Limoges, 2006; Mortola and Sequin, 2009; Fahlman et al., 2017b). A higher *f*_R_ may indicate a different breathing strategy in the offshore animals, with lower *V*_T_ to maintain the same alveolar ventilation. Still, while the average s*V*_T_ was not significantly different from previous studies of coastal ecotype bottlenose dolphins, the volumes were at the lower end and with high variability, and we may not have been able to detect a difference given our small sample size. Thus, we cannot determine whether offshore dolphins may choose to breathe more frequently with lower *V*_T_. A shallower *V*_T_ would support the suggestion that deep-diving cetaceans dive with a lower lung volume (Miller et al., 2004; Piscitelli et al., 2010), which would help minimize N_2_ uptake (Bostrom et al., 2008; Fahlman et al., 2009). A lower s*V*_T_ may indicate a lower TLC as has been suggested as a potential adaptation to reduce N_2_ uptake and risk of gas emboli in deep-diving species (Scholander, 1940; Miller et al., 2004; Bostrom et al., 2008; Piscitelli et al., 2010). Additional measurements on deep-diving dolphins may confirm this hypothesis, and alternative methods to assess respiratory capacity or diving lung volume using phonospirometry (Sumich, 2001; Sumich and May, 2009; van der Hoop et al., 2014), respiratory sinus arrhythmias, or body acceleration/movement (Fahlman et al., 2017c) may provide alternatives to explore these physiological traits for free-ranging individuals.

### Mechanisms to Avoid Lung Barotrauma

Scholander (1940) proposed that the anatomy of the respiratory system in marine mammals, with a compliant alveolar space and stiff airways, would help reduce gas exchange and the risk of decompression sickness. A number of studies have evaluated the respiratory mechanics in marine mammals and concluded that the s*C*_L_ is greater as compared with terrestrial mammals (Fahlman et al., 2017b). In a previous study, it was found that the s*C*_L_ in pinnipeds undergoing rehabilitation was higher than in animals raised under human care (Fahlman et al., 2014). One proposed explanation was that lung conditioning, by repeated diving, results in increased s*C*_L_ to reduce the risk for lung barotrauma. In human apnea divers, increased vital capacity has been reported following long-term training that included repeated expansion and compression of the thoracic space, similar to what happens during a breath-hold dive (Johansson and Schagatay, 2012). Consequently, frequent compression collapse/atelectasis and recruitment may result in more compliant chest or lung parenchyma. We therefore hypothesized that the offshore, deep-diving dolphins should exhibit a higher s*C*_L_ as compared with the dolphins that had been housed under managed care and with limited opportunity to perform deep dives. Our results indicate that the s*C*_L_ appears to be significantly higher as compared with terrestrial species, including humans, but there was no difference compared to dolphins that have lived for extended durations in zoological facilities (Fahlman et al., 2015, 2017b).

While s*C*_L_ may be an important variable to reduce lung barotrauma during diving, a compliant chest/thorax is probably more important. Chest compliance has been measured in pinnipeds, and it was shown that it was considerably more compliant than the lungs (Leith, 1976; Fahlman et al., 2014), and the flexible rib cage allows the lungs to empty to very low lung volumes. In cetaceans, on the other hand, no study has measured the compliance of the thorax in live animals at this point. It has been shown photographically that the chest in dolphins is very compressible (Ridgway et al., 1969). However, compliance measurements on stranded and deceased common (*Delphinus delphis*) and Atlantic white-sided (*Lagenorhynchus acutus*) dolphins indicate that unlike the pinnipeds, the chest is considerably stiffer than the lungs (**Figure 1**). The high s*C*_L_, and reinforced airways may help generate the high respiratory flow seen in cetaceans, and the elastic recoil may help aid compression of the lung to low volumes during diving (Kooyman and Cornell, 1973, Kooyman and Cornell, 1981; Fahlman et al., 2011, 2015, 2017b; Moore et al., 2014). While similar measurements should be repeated in live animals, these data provide an understanding about the extent that respiratory compliance has on deep-diving adaptations.

Recent work has resulted in a new hypothesis how cetaceans manage gases during diving and it has been suggested that they have specialized lung architecture, including collateral ventilation, and volitional control of autonomic cardiac responses (Fahlman et al., 2017b; García-Párraga et al., 2018). The lung architecture would help create two pulmonary regions with distinctly different levels of alveolar ventilation ($\dot{V}$_A_) and perfusion (*$\dot{Q}$*). By varying the level of ventilation-perfusion matching, the lung would be able to selectively exchange gases with different gas solubilities (West, 1962; Farhi and Yokoyama, 1967), thereby providing exchange of O_2_ and CO_2_ while minimizing N_2_ exchange (García-Párraga et al., 2018). Stressful situations could alter the ventilation-perfusion match, causing increased N_2_ exchange and risk for gas emboli. This provides avenues for new areas of research, offering an alternative explanation for how marine vertebrates can avoid the diving-related problems observed in human divers, and how failure of this adaptation may result in diving-related trauma (Frantzis, 1998; Fernandez et al., 2005, García-Párraga et al., 2014; Fernández et al., 2017; Fahlman et al., 2017a). Thus, if active management of pulmonary shunt and gas exchange is important, it makes sense that the s*C*_L_ is not different between deep-diving and shallow-diving bottlenose dolphins. Still, the hypothesis made by Scholander (1940) that there should be significant differences in the compliance between the upper and lower airways is important as it allows compression and variation of the $\dot{V}$_A_/*$\dot{Q}$* to alter exchange of the different gases (Hodanbosi et al., 2016; Fahlman et al., 2018b).

In addition, both ecotypes may have similar traits to deal with pressure changes. The largest relative difference in pressure occurs close to the surface. At a depth of 10 m, the lung volume is reduced to half that at the surface, and the change in volume decreases exponentially with depth. Thus, to prevent pulmonary barotrauma all diving marine mammals should have a compliant lung/alveolar space, and a chest that is either highly compliant or recoils inward to low volumes. Evolutionary pressure may have resulted in different solutions to a common problem. It appears that all diving marine mammals measured to date have a similar s*C*_L_, regardless of ecotype, that is greater than that measured in land mammals (Fahlman et al., 2017b). Chest compliance, on the other hand, appears to differ among orders, where measurements on live pinnipeds suggest that chest compliance is high, which allows pressure to compress the lung without resistance (Fahlman et al., 2014). In cetaceans, on the other hand, the need for high respiratory flow seems to have created a chest that is stiffer than the lung, but that recoils strongly inward to low volumes (**Figure 1**). Thus, both strategies provide a respiratory system with a lung that can compress, an alveolar space that can empty to low volumes, and vary $\dot{V}$_A_ to allow matching with *$\dot{Q}$* to allow management of gas exchange (García-Párraga et al., 2018).

## Conclusion

The measured resting s$\dot{V}$O_2_ in offshore bottlenose dolphins did not differ from measurements done for shallow-diving dolphins or measurements in other marine mammal species (Yazdi et al., 1999; Noren et al., 2013; Fahlman et al., 2015, 2017b). We showed that the offshore dolphins have greater O_2_ storage capacity (greater blood Hct), and propose that increased blood and muscle volume, and increased muscle myoglobin may also be important to increase O_2_ storage capacity. The RMR was on the low end but not different from RMR of coastal bottlenose dolphins. Thus, additional data on RMR of offshore dolphins will help resolve if increased muscle mass results in reduced basal metabolic rate in deep divers (Pabst et al., 2016). The s*C*_L_ measurements for the offshore ecotype were similar to those in shallow-diving dolphins, suggesting a lack of specialized adaptations to lung compliance. This supports the idea that since the most extreme pressure changes happen within the first 10–100 m of diving, and further specializations to enable longer and deeper dives do not add much pulmonary stress. These data suggest that the structural properties of the lung itself may not differ substantially between ecotypes within a marine mammal species and perhaps across species, but that chest compliance is most likely different in cetaceans versus pinnipeds. In both cases, the structural properties allow increasing pressure to compress the alveolar space to very low volumes, preventing barotrauma. This supports a recent hypothesis that cetaceans vary $\dot{V}$_A_/*$\dot{Q}$* to selectively manage gas exchange (García-Párraga et al., 2018). If this hypothesis is correct, we would not expect to see major differences in the s*C*_L_ between deep and shallow divers as during natural dives they would selectively control their gas exchange, making maximum dive depth per se a relatively minor determinant of s*C*_L_.

## Author Contributions

All the authors participated in the field-work and gave final approval for the publication. AF, MJM, RW, JS, and RS conceived of the study, and designed the experiments. AF, MJM, RW, JS, FHJ, RFT, and RS collected the data. AF and AA analyzed the data. AF carried out the statistical analysis, and drafted the paper, with feedback from the co-authors.

## Conflict of Interest Statement

The authors declare that the research was conducted in the absence of any commercial or financial relationships that could be construed as a potential conflict of interest.

## Abbreviations

CL: dynamic lung compliance
*f*_R_: breathing frequency
*M*_b_: body mass
s*C*_L_: mass-specific lung compliance
STPD: standard temperature pressure dry
TLC: total lung capacity
TLC_est_: estimated total lung capacity
$\dot{V}$O_2_: oxygen consumption rate
*V*_T_: tidal volume
$\dot{V}$_A_: Alveolar ventilation
*$\dot{Q}$*: Alveolar perfusion
